# Fock Space Coupled-Cluster Method for the Ground and Excited States of the NaMg^+^ Molecular Cation

**DOI:** 10.1021/acs.jpca.4c04275

**Published:** 2024-08-09

**Authors:** Grzegorz Skrzyński, Monika Musial

**Affiliations:** Institute of Chemistry, University of Silesia in Katowice, Szkolna 9, 40-006 Katowice, Poland

## Abstract

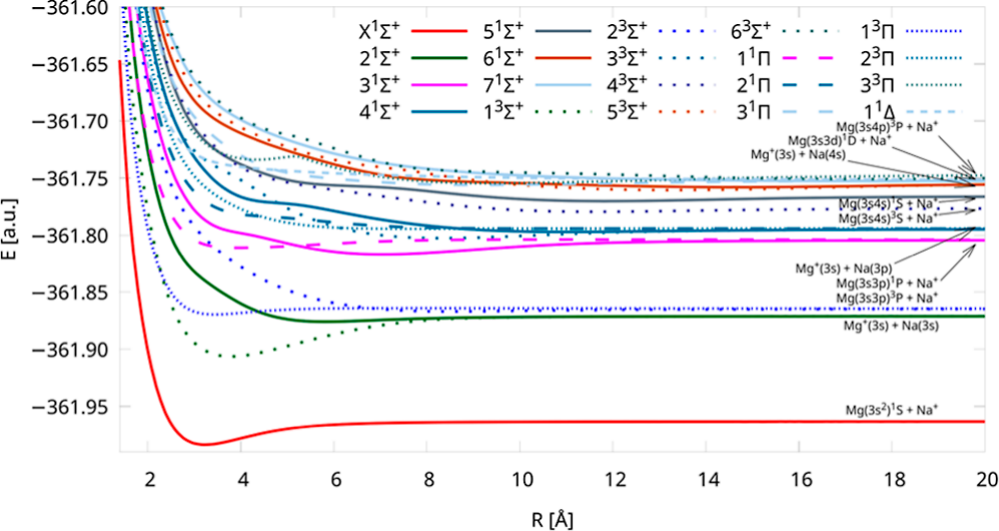

This study presents a theoretical investigation of the potential energy curves (PECs) and spectroscopic constants of the NaMg^+^ molecular cation using the intermediate Hamiltonian Fock space coupled-cluster method with singles and doubles within the (2,0) sector [IH-FS-CCSD(2,0)]. The NaMg^+^ cation has gained scientific interest due to its potential applications in ultracold chemistry, yet it remains experimentally unexplored. We computed 20 lowest-lying PECs and assigned them to 10 dissociation limits. Our results demonstrated high accuracy, and computed curves are smooth and continuous over the entire range of interatomic distances. The study validates the effectiveness of the IH-FS-CCSD(2,0) method in describing PECs of diatomic molecular cations composed of alkali and alkaline earth metals and provides a solid theoretical foundation for further studies of NaMg^+^ and similar systems.

## 1 Introduction

Studies involving diatomic molecular cations composed of alkali and alkaline earth atoms have become a rapidly growing scientific field in recent decades.^1−6^ Since these systems have a positive charge, they can be controlled by an external electric field, which is a significant advantage in experimental studies. Several researchers have explored this kind of molecular cations in ultracold conditions, including studies of Feshbach resonances of LiBa^+^,^1^ simulations of solid-state physics with a KCa^+^ cation,^2^ building atom-ion quantum gates using RbBa^+^,^3^ or the collision studies of LiCa^+^,^4^ RbCa^+^,^5^ and RbSr^+^^6^ cations, among others.

In this context, the theoretical studies of potential energy curves (PECs) of diatomics are essential as they serve as a groundwork to produce ultracold species in experimental studies. In this work, we studied PECs and spectroscopic constants of NaMg^+^, which is an example of previously mentioned molecular cations of alkali and alkaline earth metals. The earlier studies of NaMg^+^ are limited and reported only in a few papers in the context of theoretical investigations of its PECs and spectroscopic constants and, as far as we know, there are no experimental studies of the NaMg^+^ molecular cation. In the 1980s, Peyerimhoff and Bruna carried out calculations for the ground and some of the excited states of NaMg^+^ using multireference configuration interaction with singles and doubles (MRCISD).^7,8^ Then, in 2017, Fedorov et al.^9^ studied NaMg^+^ using coupled-cluster (CC)^10−32^ and MRCISD methods, and in 2020, Śmiałkowski and Tomza^33^ performed the CC calculations for this system, although both works were limited only to the ground state of NaMg^+^. Finally, in 2020, the group of Berriche^34^ conducted an extensive study of NaMg^+^ for a number of excited states using a pseudopotential approach combined with CISD, treating the system as having two electrons moving in the potential of the two atomic cores. NaMg^+^ has not yet been cooled down to ultracold temperatures due to the fact that it does not meet the criteria for the standard procedure of laser cooling.^34^ Thus, its properties are yet to be determined using experimental methods. It indicates the necessity for comprehensive theoretical studies of this molecular cation.

The molecular cations composed of alkali and alkaline earth metals are systems characterized by having two valence electrons. In this study of PECs, we decided to employ the IH-FS-CCSD (the intermediate Hamiltonian Fock space multireference CC method with singles and doubles) method, applied to the (2,0) sector.^35^ This strategy has been validated not only in the studies of diatomic molecules composed of alkali metals, e.g., Li_2_,^36^ NaLi,^37^ Na_2_,^38^ and LiRb,^39^ but also in the study of the system similar to NaMg^+^—the LiMg^+^ molecular cation.^40^ The main advantage of the method is the fact that it is a purely first-principles method, free of any additional parameters optimized for the chosen system. Moreover, IH-FS-CCSD(2,0) is a size-extensive scheme, meaning that the energies of electronic states of the studied system should converge at an infinite distance to the sum of atomic values, a crucial property in studies of PECs for distances far from the equilibrium. We also correlate all electrons of the system. The IH-FS-CCSD(2,0) strategy used in the calculations for neutral molecules (alkali metal dimers) is based on the idea that we choose a doubly ionized system as a reference, producing closed-shell fragments upon dissociation. Then, we perform calculations using the double-electron attachment (DEA) formalism and produce the energies of the ground and excited states of the studied system. Herein, we study the NaMg^+^ molecular cation; therefore, we chose as the reference system the triple-positive ion NaMg^3+^
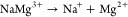
1Afterward, we used the DEA formalism
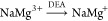
2and obtained the energies of the desired NaMg^+^ cation. The strategy presented above allows us to eliminate the problem that is still a challenge for modern computational chemistry: the proper treatment of open-shell fragments, which are produced during the process of homolytic dissociation of a single bond. The use of the restricted Hartree–Fock scheme (RHF) for bond lengths significantly distant from the equilibrium distance is improper. In this case, the unrestricted Hartree–Fock (UHF) or restricted open-shell Hartree–Fock (ROHF) method should be used. The major drawback is their problems with convergence of the HF and post-HF equations. Several solutions were proposed in the literature to address this issue. One of them is the equation-of-motion CC for excited states (EE-EOM-CC).^30,41−56^ However, even though the method is considered as “the method of choice” for studying excited states, it lacks the property of size-extensivity. Thus, its use is limited in the studies of PECs. Another more frequently used technique is the MRCI method, which is size-extensive only in its full CI (FCI) variant, not possible to realize for a multielectron system. An alternative is to replace the core electrons with a pseudopotential or an effective core potential (ECP).^34,57,58^ In this case, the electron correlation is typically considered solely for valence electrons, often limited to the CISD method, which is equivalent to the FCI for two-electron systems. This approach also requires considering some additional parameters representing the potential of core electrons, and these parameters cannot be used universally for any chosen system.

The IH-FS-CCSD(2,0) eliminates the above-mentioned problem with open-shell fragments by employing the DEA strategy, which makes it possible to use the RHF function as a reference function for any internuclear distance. Moreover, it is a strictly size-extensive ab initio method with a correlation of all electrons.

In the next section, the IH-FS-CCSD(2,0) method is described in detail.

## 2 Methods

The coupled cluster method^10−32^ is based upon the exponential expansion of a reference function
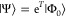
3where |Ψ⟩—exact wave function, *T*—cluster operator, and |Φ_0_⟩—reference function. The presence of the *T* operator guarantees that the correlation energy is included in the calculations, and this feature is essential to obtain the best possible accuracy with respect to experimental studies. The *T* operator is written as
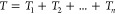
4
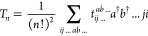
5*T*_*n*_ describes the excitation from the occupied orbitals *i*, *j*, ... into unoccupied (virtual) orbitals *a*, *b*, ... In our case, *T* = *T*_1_ + *T*_2_ for the CCSD model. The *t*_*ij*..._^*ab*...^ amplitudes are the solution of the CC equations obtained by a projection of the *H̅* (≡ e^–*T*^*H*e^*T*^) operator against excited configurations ⟨Φ_*ij*..._^*ab*...^|
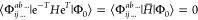
6

The principal idea of the Fock space multireference approach^59−74^ is to replace the Schrödinger equation
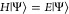
7where Ψ is the exact wave function defined within the full configurational space, *H* is the Hamiltonian, and *E* is the energy, with the equation
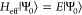
8to obtain only a few eigenvalues out of the whole spectrum to avoid the diagonalization of the *H* operator in the large configurational space. The *H*_eff_ in eq 8 is called an effective Hamiltonian. It is defined within a model space *M*, spanned by the *m* model determinants Φ_I_, with the projection operator *P*. The orthogonal space is denoted as *M*_⊥_ with projection operator *Q* (*Q* = 1 – *P*). The effective Hamiltonian operator is defined as
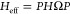
9where the wave operator Ω is used to construct the exact wave function from the model function |Ψ_0_⟩
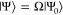
10and the latter is obtained by the action of *P* on the exact wave function
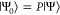
11

In the Fock space coupled cluster formalism, the Ω operator, known also as a valence universal one, is defined as
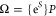
12where *S* is a cluster operator responsible for excitations from the model space to the orthogonal one and the braces indicate that normal ordering should be applied to each term of the expansion.

The characteristic feature of the Fock space formalism is that the model space is composed of the configurations obtained not only by electronic excitations within the active space but also via electron attachment and ionization processes. The model space is partitioned into sectors denoted, in our case, by (*m*, 0), which indicate the number of electrons that have been added to the reference system. Hence, the one-dimensional (0,0) sector is associated with the ground state, the (1,0) sector with single-electron-attached states, the (2,0) sector with double electron-attached states, etc. The orbital levels are divided into active levels, which change occupation, and inactive levels, for which the occupation is constant for all reference determinants. The other characteristic feature of the FS approach is the hierarchical structure of the coupled cluster solutions. This implies that when we formulate the Fock space problem for this sector (*m*, 0), then the cluster operator includes all lower-rank sectors. Herein, only the (2,0) sector of the model space will be applied in order to calculate the PECs of the studied molecular cation. A general expression for the *H*_eff_ operator for this sector at the CCSD level can be written out as
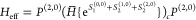
13with the model space projection operator *P*^(2,0)^ defined as
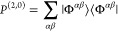
14where Φ^αβ^ denotes the additional electrons placed on the virtual levels α and β and the respective orthogonal space projector *Q*^(2,0)^ = 1 – *P*^(2,0)^.

A serious obstacle in implementing the FS-CC method was the so-called intruder state problem.^75,76^ It causes severe difficulties with convergence. To avoid these problems, the intermediate Hamiltonian (IH) approach was introduced.^35,77−85^ Briefly, the main idea of IH is to introduce a buffer space between the desired states and the others. This is achieved by constructing the *H*_I_ operator using the *H̅* and the cluster operators *S* known from the (1,0) sector
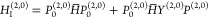
15with
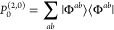
16and the wave operators *Y*^(2,0)^ and *X*^(2,0)^ are defined as
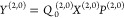
17
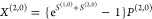
18with
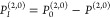
19
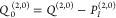
20

It should also be mentioned that the Fock space approach at the (1,0) sector is equivalent to the EOM-CC scheme applied to the electron affinity. This equivalence means that the eigenvalues are identical, while the eigenvectors can be obtained from each other by a simple transformation. Thus, for the (1,0) sector, we have
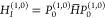
21

## 3 Results and Discussion

The IH-FS-CCSD(2,0)^35^ calculations were performed using ACES II^86^ ver. 2.7.0 software package augmented with our own local module. All spectroscopic constants were determined using the 8.0 version of Robert J. Le Roy’s LEVEL program.^87^ In all calculations, the uncontracted ANO-RCC^88^ basis set with additional diffuse functions was used—we refer to this basis set as unANO-RCC+. The six additional exponents for the Na atom are as follows: 0.0044652, 0.0017860 for the s shell; 0.0029282, 0.0011713 for the p shell; and 0.0221083, 0.0088459 for the d shell. For the Mg atom, added exponents for the diffuse functions are the following: 0.0062002, 0.0024801 for the s shell; 0.0053322, 0.0021329 for the p shell; and 0.0209515, 0.0083806 for the d shell. The new exponents were determined with the even-tempered scheme,^89^ and the ratio between consecutive exponents is equal to 0.4 for both atoms. The augmented basis set yields the correct ordering of the atomic electronic states. The resulting unANO-RCC+ basis set is composed of 275 spherical harmonic polarization functions. The NaMg^3+^ cation was chosen as the reference system for IH-FS-CCSD(2,0) calculations. The reference function employed throughout the study was always obtained by the RHF method. All of the electrons were correlated. The active space size for IH-FS-CCSD(2,0)/unANO-RCC+ calculations was set to 83 (i.e., the 83 lowest virtual orbitals chosen as active), resulting in a model space size of 6889.

The IH-FS-CCSD(2,0) method possesses the property that is crucial in the calculations of PECs, i.e., the size-extensivity. This property states that the energies of the electronic states of a system must converge to the sum of their atomic values at an infinite distance. In Table 1, we presented the following computed values: the first column indicates the dissociation limits, the next four columns indicate the atomic/ionic structure and give their energy values, the next one displays the sum of these energies, and the last column presents the energy at the corresponding dissociation limit obtained using the IH-FS-CCSD(2,0) method. The energies of the Na^+^ cation were obtained by using the CCSD method (no electrons attached). For the Na atom and the Mg^+^ cation, IH-FS-CCSD(1,0) (≡EA-EOM-CCSD) was used (the single electron attachment). For the Mg atom, the IH-FS-CCSD(2,0) method was utilized (the double electron attachment). The last two columns show equal values, which proves the size-extensivity of the method.

**Table 1:** Energies of Electronic States at the Dissociation Limit of the NaMg^+^ Molecular Cation Compared to Respective Atomic Energies

diss. limit	Na/Na^+^		Mg/Mg^+^		Na/Na^+^+Mg/Mg^+^	NaMg^+^ R = ∞
	IH-FS-CCSD(1,0)/CCSD		IH-FS-CCSD(2,0)/IH-FS-CCSD(1,0)			IH-FS-CCSD(2,0)
	Config	*E* (a.u.)	Config	*E* (a.u.)	*E* (a.u.)	*E* (a.u.)
Mg(3s^2^)^1^S + Na^+^	[Ne]	–161.955463	[Ne] (3s^2^)^1^S	–200.007795	–361.963258	–361.963258
Mg^+^(3s) + Na(3s)	[Ne] 3s	–161.143457	[Ne] 3s	–199.727651	–360.871108	–361.871108
Mg(3s3p)^3^P + Na^+^	[Ne]	–161.955463	[Ne] (3s3p)^3^P	–199.909041	–361.864504	–361.864504
Mg(3s3p)^1^P + Na^+^	[Ne]	–161.955463	[Ne] (3s3p)^1^P	–199.848623	–361.804086	–361.804086
Mg^+^(3s) + Na(3p)	[Ne] 3p	–162.066733	[Ne] 3s	–199.727651	–361.794384	–361.794384
Mg(3s4s)^3^S + Na^+^	[Ne]	–161.955463	[Ne] (3s4s)^3^S	–199.820787	–361.776250	–361.776250
Mg(3s4s)^1^S + Na^+^	[Ne]	–161.955463	[Ne] (3s4s)^1^S	–199.810343	–361.765806	–361.765806
Mg^+^(3s) + Na(4s)	[Ne] 4s	–162.026846	[Ne] 3s	–199.727651	–361.754497	–361.754497
Mg(3s3d)^1^D + Na^+^	[Ne]	–161.955463	[Ne] (3s3d)^1^D	–199.796326	–361.751789	–361.751789
Mg(3s4p)^3^P + Na^+^	[Ne]	–161.955463	[Ne] (3s4p)^3^P	–199.790617	–361.746080	–361.746080

**Figure 1:**
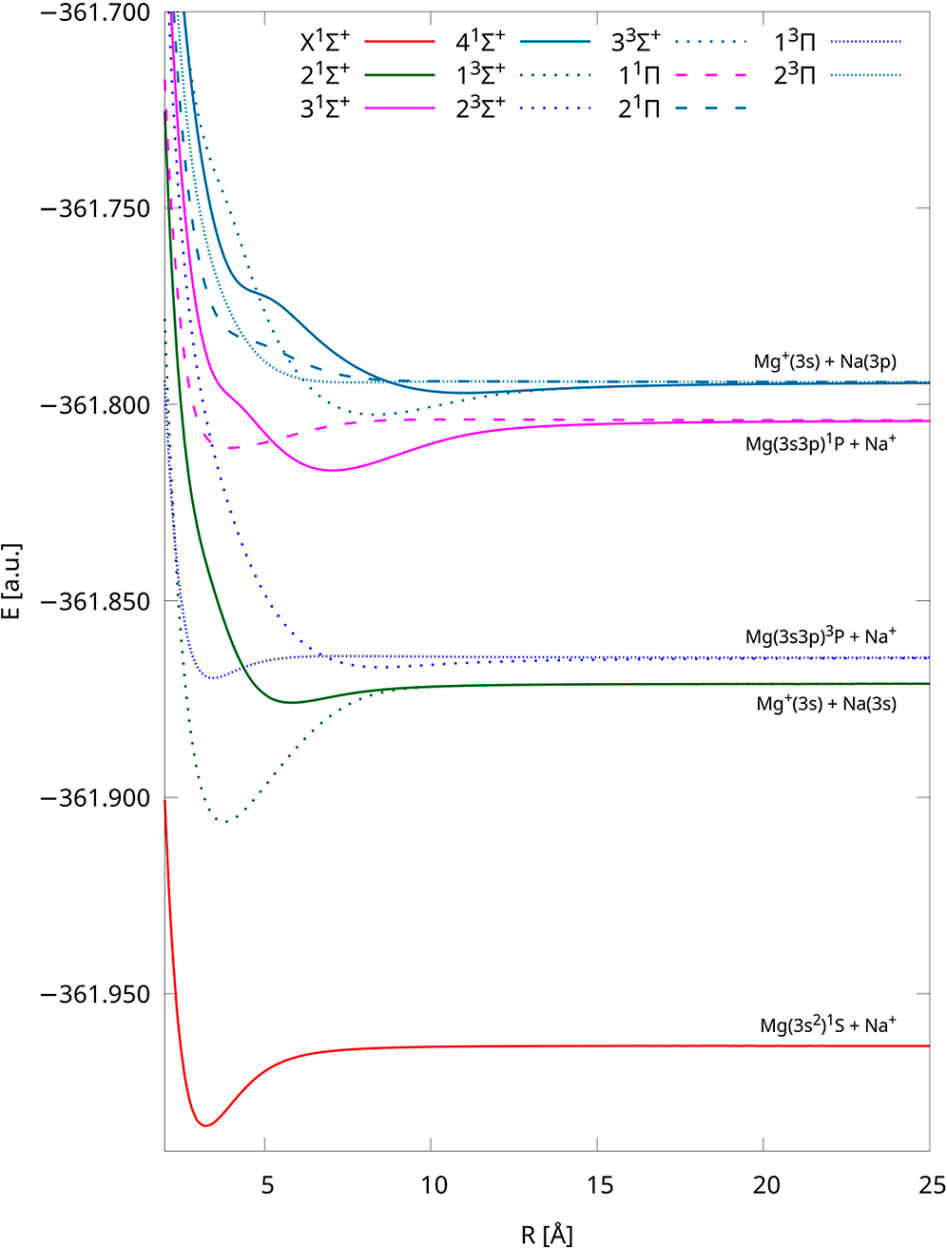
Potential energy curves of NaMg^+^ calculated using the IH-FS-CCSD(2,0)/unANO-RCC+ method for the five lowest dissociation limits.

**Figure 2:**
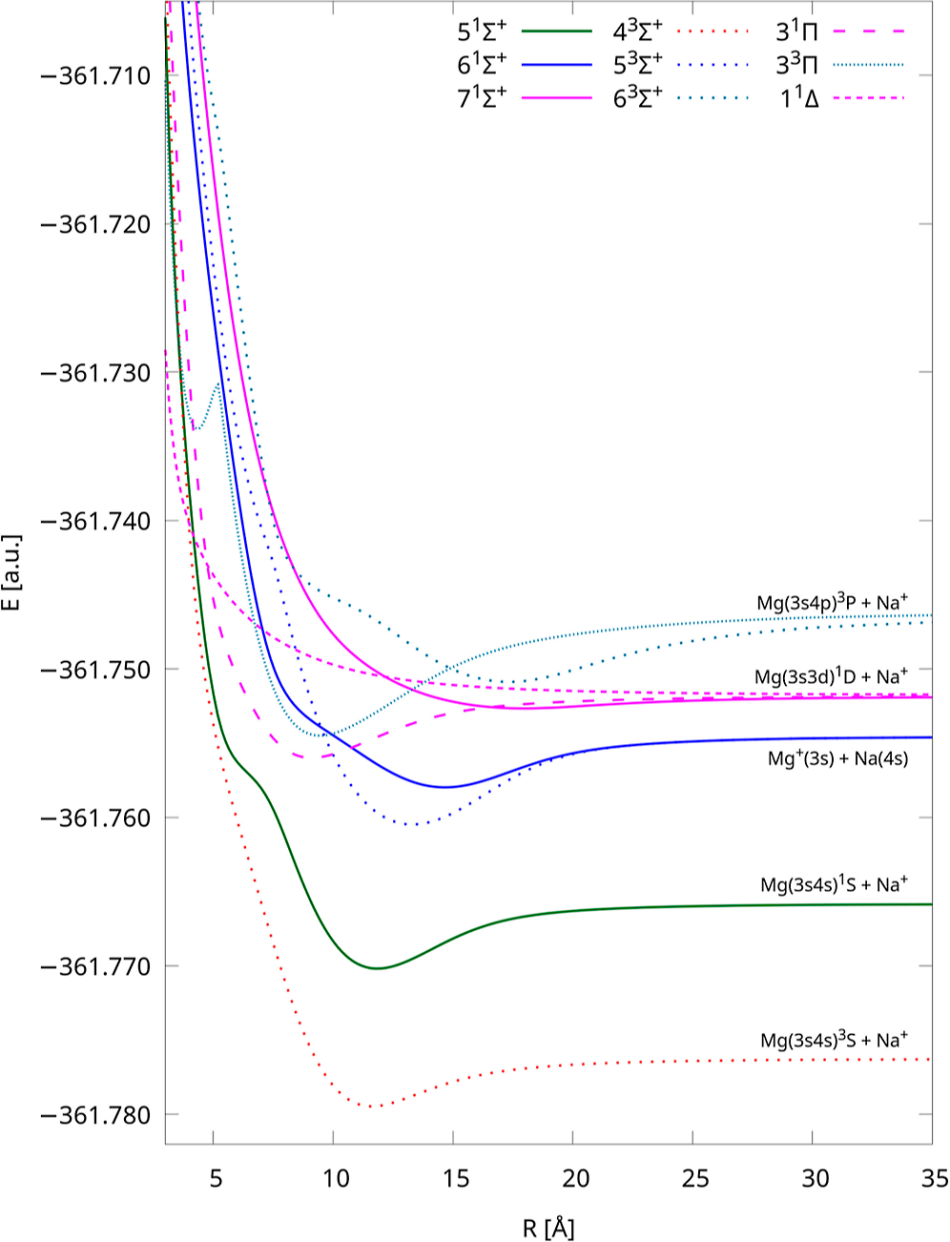
Potential energy curves of NaMg^+^ calculated using the IH-FS-CCSD(2,0)/unANO-RCC+ method for the next five dissociation limits.

We computed the 20 lowest-lying potential energy curves of the NaMg^+^ molecular cation using the IH-FS-CCSD(2,0)/unANO-RCC+ method. These PECs were assigned to 10 distinct dissociation limits. In order to improve the visibility of unique curves, we divided them into Figures 1 and 2—the first one depicts the five lowest dissociation limits of NaMg^+^, and the second one shows the next five ones. The PECs have different colors for each dissociation limit as well as five different point types for each symmetry and multiplicity of the electronic state. In Figure 1, the PECs are limited to 25 Å, and they are limited to 35 Å in Figure 2, but the energies up to 500 Å are available in the Supporting Information. Obtained curves are smooth over the whole range of interatomic distances from the equilibrium to the dissociation limit.

As we mentioned in the Introduction section, only the group of Berriche et al.^34^ conducted the study for the significant number of PECs of NaMg^+^; thus, we will mostly compare our findings with theirs. In general, the shapes of PECs are similar to those presented in ref (34) along with characteristic undulations and distortions from the typical Morse-like profile. These atypical shapes are the result of avoided crossings of neighboring curves of the same multiplicity and symmetry, which originate from the interactions and charge transfer processes between the electronic states of Na–Mg^+^ and Na^+^–Mg. The avoided-crossing positions were compared with those from ref (34) and they show a high coincidence–the average difference is less than 1% (Table 2).

**Table 2:** Avoided-Crossing Positions of the NaMg^+^ Molecular Cation Obtained Using the IH-FS-CCSD(2,0)/unANO-RCC+ Method

states	position in this work (Å)
3^1^Σ^+^/4^1^Σ^+^	4.24
	13.60
4^1^Σ^+^/5^1^Σ^+^	2.94
	5.29
	12.57
5^1^Σ^+^/6^1^Σ^+^	7.68
	16.43
6^1^Σ^+^/7^1^Σ^+^	5.53
	26.08
5^3^Σ^+^/6^3^Σ^+^	4.24
	7.60
	19.57

We also find that only one among the 20 lowest-lying PECs has two potential wells, i.e., the 3^3^Π state. It agrees with the curves computed in ref (34).

The calculated PECs were used to extract the spectroscopic constants of NaMg^+^, i.e., equilibrium distances *R*_e_, well depths *D*_e_, adiabatic excitation energies *T*_e_, harmonic frequencies ω_e_, anharmonicity constants ω_e_*x*_e_, and equilibrium rotational constants *B*_e_. The results are gathered in Table 3 and compared with the available theoretical calculations: MRCISD values from refs (7 and 8), CCSDT and MRCI results from ref (9), CCSD(T) value of ref (33), and finally, the results from ref (34) obtained using the pseudopotential-based method. As we mentioned earlier, there are no experimental data on the spectroscopic constants of the NaMg^+^ molecular cation.

**Table 3:** Spectroscopic Constants of NaMg^+^^g^

sym	*D*_e_	*T*_e_	*R*_e_	ω_e_	ω_e_*x*_e_	*B*_e_	source
Mg(3s^2^)^1^S + Na^+^							
X^1^Σ^+^	4490		3.233	150.88	1.13	0.137	this work^a^
	4883		3.33				(7)^b^
	3804		3.43				(8)^b^
	4522.9		3.23	150.9	1.2		(9)^c^
	4452.8		3.25	149.3	1.2		(9)^d^
	4517		3.22	151		0.138	(33)^e^
	4546		3.22	150.85	1.77	0.137280	(34)^f^
Mg^+^(3s) + Na(3s)							
2^1^Σ^+^	1059	23,656	5.815	51.37	0.62	0.042	this work^a^
	1338		5.78				(7)^b^
	1226		6.13				(8)^b^
	1239	23,665	5.77	2.23	0.96	0.042871	(34)^f^
1^3^Σ^+^	7723	16,991	3.791	120.76	0.41	0.100	this work^a^
	7797		3.94				(7)^b^
	7961		3.87				(8)^b^
	7778	17,127	3.78	121.04	0.93	0.099907	(34)^f^
Mg(3s3p)^3^P + Na^+^							
2^3^Σ^+^	527	25,637	8.507	26.92	0.47	0.020	this work^a^
	repulsive						(7)^b^
	575		8.29				(8)^b^
	639	25,901	8.30	27.70	0.08	0.02719	(34)^f^
1^3^Π	1129	25,035	3.445	100.77	1.82	0.121	this work^a^
	1798		3.57				(7)^b^
	1262		3.71				(8)^b^
	1121	25,420	3.44	98.54	2.07	0.120449	(34)^f^
Mg(3s3p)^1^P + Na^+^							
3^1^Σ^+^	2801	36,623	7.040	46.78	0.15	0.029	this work^a^
	3126		7.18				(8)^b^
	3067	36,798	7.05	45.40	0.05	0.02805	(34)^f^
1^1^Π	1541	37,883	3.938	54.14	0.34	0.093	this work^a^
	1992		4.02				(7)^b^
	1249		4.00				(8)^b^
	1622	38,244	4.02	46.92	0.45	0.086284	(34)^f^
Mg^+^(3s) + Na(3p)							
4^1^Σ^+^	599	40,955	11.020	21.24	0.24	0.012	this work^a^
	740		10.51				(8)^b^
	685	41,185	10.97	22.14	0.27	0.011868	(34)^f^
3^3^Σ^+^	1811	39,742	8.331	37.40	0.12	0.021	this work^a^
	2017	39,853	8.27	3893	0.57	0.020857	(34)^f^
2^1^Π	repulsive						this work^a^
	repulsive						(7)^b^
	81		10.24				(8)^b^
	77	41,795	9.62	10.14	1.78	0.015641	(34)^f^
2^3^Π	10	41,543	7.611	14.91	0.76	0.025	this work^a^
	122		7.58				(7)^b^
	172		7.58				(8)^b^
	207	41,665	7.51	18.49	2.16	0.025287	(34)^f^
Mg(3s4s)^3^S + Na^+^							
4^3^Σ^+^	705	44,828	11.622	22.30	0.17	0.011	this work^a^
	759	44,942	11.52	22.48	0.11	0.010750	(34)^f^
Mg(3s4s)^1^S + Na^+^							
5^1^Σ^+^	961	46,865	11.836	23.02	0.11	0.010	this work^a^
	1013	47,005	11.80	23.36	0.30	0.010259	(34)^f^
Mg^+^(3s) + Na(4s)							
6^1^Σ^+^	762	49,545	14.667	15.78	0.06	0.007	this work^a^
	920	49,725	14.17	16.07	0.17	0.007103	(34)^f^
5^3^Σ^+^	1311	48,996	13.308	20.24	0.03	0.008	this work^a^
	1546	49,099	12.98	20.49	0.10	0.008475	(34)^f^
Mg(3s3d)^1^D + Na^+^							
7^1^Σ^+^	190	50,712	17.943	7.18	0.07	0.004	this work^a^
	258	50,934	17.91	6.77	1.30	0.004463	(34)^f^
3^1^Π	920	49,982	9.100	21.69	0.12	0.017	this work^a^
	1450	49,743	9.32	25.56	0.10	0.016435	(34)^f^
1^1^Δ	repulsive						this work^a^
	repulsive						(34)^f^
Mg(3s4p)^3^P + Na^+^							
6^3^Σ^+^	1050	51,105	17.340	13.56	0.04	0.005	this work^a^
	1194	51,306	17.36	14.19	0.50	0.004714	(34)^f^
3^3^Π 1st min	–2680	54,835	4.330	100.67	1.28	0.077	this work^a^
	519	55,510	4.33	95.93	0.56	0.076224	(34)^f^
3^3^Π 2nd min	1845	50,310	9.445	25.41	0.10	0.016	this work^a^
	2562	49,948	9.462	28.93	0.56	0.076224	(34)^f^

a The method used in this work, which is IH-FS-CCSD(2,0)/unANO-RCC+, as described in detail in the text.

b The method used in refs (7 and 8) is MRCISD.

c The first method used in ref (9) is CCSDT/cc-pCVQZ.

d The second method used in ref (9) is MRCI/cc-pCVQZ.

e The method used in ref (33) is CCSD(T)/aug-cc-pCVQZ.

f The method used in ref (34) is based on pseudopotentials. The basis set for sodium was (7s6p5d3*f*/6s5p4d2f); for magnesium, it was (9s7p5d4*f*/7s7p4d4f).

g *D*_e_, *T*_e_, ω_e_, ω_e_*x*_e_, and *B*_e_ are given in cm^–1^; *R*_e_ is given in Å.

Since the results from ref (34) are the only ones available for all of the electronic states of NaMg^+^ calculated in this work, and also, it is the most recent literature data for this molecular cation, we will discuss our values mostly in comparison with this paper. The agreement between our values and those from ref (34) depends on the spectroscopic constant and on the considered electronic state.

We identified two states as repulsive, i.e., 2^1^Π and 1^1^Δ. As of the first one, it contradicts the findings in refs (8 and 34) but their *D*_e_ values are very small (81 and 77 cm^–1^, respectively); thus, we consider these are possibly plateaus. The second one agrees with ref (34), where this state is also described as the repulsive one.

Generally, we see good agreement in the values of equilibrium distances. The close correspondence of *R*_e_ implicates a respective agreement in the derived *B*_e_ values. As for *T*_e_ values, the agreement is the best and most consistent. The largest error of ∼1.5% is seen for the 1^3^Π state, but for the majority of states, it is less than 1%.

The agreement with ref (34) in the obtained ω_e_ values is diverse. For some states, we see a very close match (e.g., for the X^1^Σ^+^ and 4^3^Σ^+^ states), but for some others, we observe larger differences (cf 2^1^Σ^+^ and 3^3^Σ^+^). We have no explanation for these discrepancies, given that the shapes of our PECs are similar to those in ref (34). Analogously larger differences between our results and those of ref (34) occur in the case of the ω_e_*x*_e_ constant. The number of states where these values are close is limited, e.g., 4^1^Σ^+^ or 3^1^Π. To sum up, the agreement with ref (34) is in most cases satisfactory, with minor deviations in some electronic states.

## 4 Conclusions

In this study, we explored the PECs and spectroscopic constants of the NaMg^+^ molecular cation using the IH-FS-CCSD(2,0) method and the unANO-RCC+ basis set. To address the challenge of an accurate description of the dissociation of closed-shell species into open-shell parts, we employed the double-electron attachment formalism and the RHF reference function. For the first time, the fully size-extensive first-principles method with correlation of all electrons was used to calculate PECs for the entire range of interatomic distances. The curves of the 20 lowest-lying electronic states of NaMg^+^ were obtained and assigned to 10 dissociation limits. The PECs were used to determine the spectroscopic constants of NaMg^+^: equilibrium distances *R*_e_, well depths *D*_e_, adiabatic excitation energies *T*_e_, harmonic frequencies ω_e_, anharmonicity constants ω_e_*x*_e_, and equilibrium rotational constants *B*_e_. Our results demonstrate good agreement with those of earlier theoretical work.^34^

Once more, we were able to prove the usefulness of the IH-FS-CCSD(2,0) method in the studies of diatomic systems with two valence electrons. We expect that the results for NaMg^+^ can be considered as benchmark values based on the outcomes of previous studies that utilized this approach in calculations of PECs and spectroscopic constants.^37−40^ The experimental validation of our theoretical results would be a crucial next step, particularly in the context of ultracold chemistry and collision studies. The broad area of potential applications for this type of molecular cation, such as in the investigation of quantum computing, simulations in the solid-state physics, or precise measurements, holds significant importance for future endeavors.

## Supporting Information Available

Total IH-FS-CCSD(2,0)/unANO-RCC+ energies of NaMg^+^ for all computed states (PDF)
