# Supplementary material for: Fock Space Coupled-Cluster Method for the Ground and Excited States of the NaMg+ Molecular Cation
Source: J Phys Chem A. 2024 Aug 9;128(33):6972–80. doi: 10.1021/acs.jpca.4c04275 (PMC11345837; doi:10.1021/acs.jpca.4c04275)
Supplement: Supplementary file 1 — jp4c04275_si_001.pdf [file jp4c04275_si_001.pdf]

Supplementary Material for

The Fock Space Coupled-Cluster Method for the Ground and Excited States of the $NaMg^+$ Molecular Cation

Grzegorz Skrzyński*, Monika Musial†

Institute of Chemistry

University of Silesia in Katowice

Szkolna 9, 40-006 Katowice, Poland

August 1, 2024

*Corresponding author: e-mail address: grzegorz.skrzynski@us.edu.pl

†Corresponding author: e-mail address: monika.musial@us.edu.pl

Table S1: Total IH-FS-CCSD (2,0)/unANO-RCC+ energies for the $X^1\Sigma^+$ state of $NaMg^+$.

R (Å)	E (a.u.)	R (Å)	E (a.u.)	R (Å)	E (a.u.)	R (Å)	E (a.u.)
1.4	-361.646668	6.6	-361.965028	11.6	-361.963410	17.0	-361.963289
1.6	-361.775644	6.8	-361.964800	11.8	-361.963400	17.2	-361.963288
1.8	-361.850495	7.0	-361.964608	12.0	-361.963390	17.4	-361.963286
2.0	-361.900626	7.2	-361.964447	12.2	-361.963381	17.6	-361.963285
2.2	-361.934797	7.4	-361.964309	12.4	-361.963373	18.0	-361.963283
2.4	-361.957206	7.6	-361.964192	12.6	-361.963366	19.0	-361.963278
2.6	-361.970999	7.8	-361.964090	12.8	-361.963359	20.0	-361.963274
2.8	-361.978770	8.0	-361.964003	13.0	-361.963353	21.0	-361.963271
3.0	-361.982523	8.2	-361.963926	13.2	-361.963347	22.0	-361.963268
3.2	-361.983696	8.4	-361.963859	13.4	-361.963341	23.0	-361.963267
3.4	-361.983276	8.6	-361.963800	13.6	-361.963336	24.0	-361.963265
3.6	-361.981929	8.8	-361.963748	13.8	-361.963332	25.0	-361.963264
3.8	-361.980104	9.0	-361.963703	14.0	-361.963327	26.0	-361.963263
4.0	-361.978099	9.2	-361.963662	14.2	-361.963323	27.0	-361.963263
4.2	-361.976103	9.4	-361.963626	14.4	-361.963320	28.0	-361.963262
4.4	-361.974231	9.6	-361.963594	14.6	-361.963316	29.0	-361.963262
4.6	-361.972545	9.8	-361.963567	14.8	-361.963313	30.0	-361.963261
4.8	-361.971071	10.0	-361.963541	15.0	-361.963310	50.0	-361.963259
5.2	-361.968746	10.2	-361.963519	15.2	-361.963308	100.0	-361.963258
5.4	-361.967865	10.4	-361.963498	15.4	-361.963305	200.0	-361.963258
5.6	-361.967136	10.6	-361.963480	15.6	-361.963302	500.0	-361.963258
5.8	-361.966535	10.8	-361.963463	15.8	-361.963300		
6.0	-361.966042	11.0	-361.963448	16.0	-361.963298		
6.2	-361.965637	11.2	-361.963435	16.6	-361.963292		
6.4	-361.965304	11.4	-361.963422	16.8	-361.963291		

Table S2: Total IH-FS-CCSD (2,0)/unANO-RCC+ energies for the $2^1\Sigma^+$ state of $NaMg^+$.

R (Å)	E (a.u.)	R (Å)	E (a.u.)	R (Å)	E (a.u.)	R (Å)	E (a.u.)
1.4	-361.465391	6.6	-361.875120	11.6	-361.871501	17.0	-361.871189
1.6	-361.596415	6.8	-361.874796	11.8	-361.871473	17.2	-361.871186
1.8	-361.672746	7.0	-361.874470	12.0	-361.871448	17.4	-361.871182
2.0	-361.725423	7.2	-361.874155	12.2	-361.871425	17.6	-361.871179
2.2	-361.763689	7.4	-361.873858	12.4	-361.871404	18.0	-361.871173
2.4	-361.791001	7.6	-361.873584	12.6	-361.871385	19.0	-361.871160
2.6	-361.809926	7.8	-361.873334	12.8	-361.871367	20.0	-361.871150
2.8	-361.822879	8.0	-361.873108	13.0	-361.871351	21.0	-361.871143
3.0	-361.832002	8.2	-361.872906	13.2	-361.871336	22.0	-361.871137
3.2	-361.838983	8.4	-361.872727	13.4	-361.871322	23.0	-361.871132
3.4	-361.844941	8.6	-361.872567	13.6	-361.871310	24.0	-361.871129
3.6	-361.850409	8.8	-361.872425	13.8	-361.871298	25.0	-361.871126
3.8	-361.855491	9.0	-361.872300	14.0	-361.871287	26.0	-361.871123
4.0	-361.860097	9.2	-361.872188	14.2	-361.871277	27.0	-361.871121
4.2	-361.864113	9.4	-361.872090	14.4	-361.871268	28.0	-361.871119
4.4	-361.867475	9.6	-361.872002	14.6	-361.871259	29.0	-361.871118
4.6	-361.870176	9.8	-361.871924	14.8	-361.871251	30.0	-361.871117
4.8	-361.872254	10.0	-361.871854	15.0	-361.871243	50.0	-361.871110
5.2	-361.874826	10.2	-361.871792	15.2	-361.871236	100.0	-361.871109
5.4	-361.875479	10.4	-361.871736	15.4	-361.871229	200.0	-361.871109
5.6	-361.875823	10.6	-361.871686	15.6	-361.871223	500.0	-361.871109
5.8	-361.875931	10.8	-361.871641	15.8	-361.871217		
6.0	-361.875867	11.0	-361.871601	16.0	-361.871212		
6.2	-361.875684	11.2	-361.871564	16.6	-361.871198		
6.4	-361.875424	11.4	-361.871531	16.8	-361.871193		

Table S3: Total IH-FS-CCSD (2,0)/unANO-RCC+ energies for the $3^1\Sigma^+$ state of $NaMg^+$.

R (Å)	E (a.u.)	R (Å)	E (a.u.)	R (Å)	E (a.u.)	R (Å)	E (a.u.)
1.4	-361.400761	6.6	-361.816481	11.6	-361.807019	17.0	-361.804701
1.6	-361.533166	6.8	-361.816744	11.8	-361.806781	17.2	-361.804676
1.8	-361.609812	7.0	-361.816847	12.0	-361.806569	17.4	-361.804652
2.0	-361.662639	7.2	-361.816807	12.2	-361.806379	17.6	-361.804630
2.2	-361.701399	7.4	-361.816643	12.4	-361.806210	18.0	-361.804588
2.4	-361.729993	7.6	-361.816374	12.6	-361.806060	19.0	-361.804502
2.6	-361.751030	7.8	-361.816017	12.8	-361.805925	20.0	-361.804435
2.8	-361.766505	8.0	-361.815591	13.0	-361.805804	21.0	-361.804381
3.0	-361.777809	8.2	-361.815108	13.2	-361.805695	22.0	-361.804338
3.2	-361.785825	8.4	-361.814583	13.4	-361.805596	23.0	-361.804303
3.4	-361.791188	8.6	-361.814028	13.6	-361.805506	24.0	-361.804274
3.6	-361.794559	8.8	-361.813446	13.8	-361.805424	25.0	-361.804250
3.8	-361.796682	9.0	-361.812859	14.0	-361.805348	26.0	-361.804230
4.0	-361.798254	9.2	-361.812270	14.2	-361.805278	27.0	-361.804213
4.2	-361.799773	9.4	-361.811689	14.4	-361.805214	28.0	-361.804198
4.4	-361.801466	9.6	-361.811122	14.6	-361.805155	29.0	-361.804186
4.6	-361.803343	9.8	-361.810583	14.8	-361.805100	30.0	-361.804175
4.8	-361.805307	10.0	-361.810058	15.0	-361.805050	50.0	-361.804103
5.2	-361.809080	10.2	-361.809563	15.2	-361.805010	100.0	-361.804088
5.4	-361.810755	10.4	-361.809098	15.4	-361.804966	200.0	-361.804086
5.6	-361.812237	10.6	-361.808667	15.6	-361.804925	500.0	-361.804086
5.8	-361.813508	10.8	-361.808271	15.8	-361.804887		
6.0	-361.814563	11.0	-361.807909	16.0	-361.804851		
6.2	-361.815405	11.2	-361.807581	16.6	-361.804755		
6.4	-361.816040	11.4	-361.807285	16.8	-361.804727		

Table S4: Total IH-FS-CCSD (2,0)/unANO-RCC+ energies for the $4^1\Sigma^+$ state of $NaMg^+$.

R (Å)	E (a.u.)	R (Å)	E (a.u.)	R (Å)	E (a.u.)	R (Å)	E (a.u.)
1.4	-361.341693	6.6	-361.783444	11.6	-361.797007	17.0	-361.795170
1.6	-361.476585	6.8	-361.784887	11.8	-361.796939	17.2	-361.795138
1.8	-361.556359	7.0	-361.786245	12.0	-361.796860	17.4	-361.795108
2.0	-361.611506	7.2	-361.787512	12.2	-361.796773	17.6	-361.795080
2.2	-361.651412	7.4	-361.788686	12.4	-361.796680	18.0	-361.795027
2.4	-361.680531	7.6	-361.789768	12.6	-361.796585	19.0	-361.794917
2.6	-361.702035	7.8	-361.790759	12.8	-361.796489	20.0	-361.794831
2.8	-361.718446	8.0	-361.791663	13.0	-361.796394	21.0	-361.794762
3.0	-361.731515	8.2	-361.792483	13.2	-361.796301	22.0	-361.794707
3.2	-361.742165	8.4	-361.793221	13.4	-361.796210	23.0	-361.794663
3.4	-361.750792	8.6	-361.793882	13.6	-361.796123	24.0	-361.794626
3.6	-361.757611	8.8	-361.794478	13.8	-361.796039	25.0	-361.794595
3.8	-361.762790	9.0	-361.794997	14.0	-361.795959	26.0	-361.794569
4.0	-361.766487	9.2	-361.795448	14.2	-361.795883	27.0	-361.794548
4.2	-361.768904	9.4	-361.795837	14.4	-361.795810	28.0	-361.794529
4.4	-361.770342	9.6	-361.796166	14.6	-361.795742	29.0	-361.794514
4.6	-361.771175	9.8	-361.796433	14.8	-361.795677	30.0	-361.794500
4.8	-361.771768	10.0	-361.796655	15.0	-361.795616	50.0	-361.794407
5.2	-361.773235	10.2	-361.796828	15.2	-361.795559	100.0	-361.794387
5.4	-361.774342	10.4	-361.796957	15.4	-361.795504	200.0	-361.794384
5.6	-361.775679	10.6	-361.797044	15.6	-361.795454	500.0	-361.794384
5.8	-361.777176	10.8	-361.797094	15.8	-361.795406		
6.0	-361.778755	11.0	-361.797112	16.0	-361.795360		
6.2	-361.780354	11.2	-361.797102	16.6	-361.795240		
6.4	-361.781927	11.4	-361.797069	16.8	-361.795204		

Table S5: Total IH-FS-CCSD (2,0)/unANO-RCC+ energies for the $5^1\Sigma^+$ state of $NaMg^+$.

R (Å)	E (a.u.)	R (Å)	E (a.u.)	R (Å)	E (a.u.)	R (Å)	E (a.u.)
1.4	-361.321457	6.6	-361.757266	11.6	-361.770160	17.0	-361.767000
1.6	-361.452822	6.8	-361.757617	11.8	-361.770184	17.2	-361.766923
1.8	-361.526377	7.0	-361.758028	12.0	-361.770174	17.4	-361.766852
2.0	-361.577448	7.2	-361.758522	12.2	-361.770134	17.6	-361.766787
2.2	-361.617774	7.4	-361.759111	12.4	-361.770068	18.0	-361.766671
2.4	-361.649558	7.6	-361.759793	12.6	-361.769979	19.0	-361.766451
2.6	-361.674063	7.8	-361.760553	12.8	-361.769870	20.0	-361.766302
2.8	-361.692530	8.0	-361.761365	13.0	-361.769745	21.0	-361.766196
3.0	-361.706106	8.2	-361.762203	13.2	-361.769606	22.0	-361.766119
3.2	-361.716013	8.4	-361.763043	13.4	-361.769458	23.0	-361.766061
3.4	-361.723410	8.6	-361.763865	13.6	-361.769301	24.0	-361.766017
3.6	-361.729183	8.8	-361.764652	13.8	-361.769139	25.0	-361.765982
3.8	-361.733913	9.0	-361.765399	14.0	-361.768974	26.0	-361.765954
4.0	-361.737945	9.2	-361.766096	14.2	-361.768807	27.0	-361.765932
4.2	-361.741472	9.4	-361.766741	14.4	-361.768641	28.0	-361.765914
4.4	-361.744590	9.6	-361.767329	14.6	-361.768478	29.0	-361.765899
4.6	-361.747331	9.8	-361.767859	14.8	-361.768318	30.0	-361.765886
4.8	-361.749688	10.0	-361.768330	15.0	-361.768163	50.0	-361.765816
5.2	-361.753178	10.2	-361.768743	15.2	-361.768015	100.0	-361.765807
5.4	-361.754341	10.4	-361.769099	15.4	-361.767873	200.0	-361.765806
5.6	-361.755189	10.6	-361.769399	15.6	-361.767737	500.0	-361.765806
5.8	-361.755803	10.8	-361.769647	15.8	-361.767609		
6.0	-361.756260	11.0	-361.769843	16.0	-361.767489		
6.2	-361.756624	11.2	-361.769991	16.6	-361.767174		
6.4	-361.756946	11.4	-361.770094	16.8	-361.767084		

Table S6: Total IH-FS-CCSD (2,0)/unANO-RCC+ energies for the $6^1\Sigma^+$ state of $NaMg^+$.

R (Å)	E (a.u.)	R (Å)	E (a.u.)	R (Å)	E (a.u.)	R (Å)	E (a.u.)
1.4	-361.297421	6.6	-361.743822	11.6	-361.756178	17.0	-361.757142
1.6	-361.431844	6.8	-361.745489	11.8	-361.756382	17.2	-361.757027
1.8	-361.509943	7.0	-361.746970	12.0	-361.756578	17.4	-361.756911
2.0	-361.563223	7.2	-361.748256	12.2	-361.756765	17.6	-361.756796
2.2	-361.602938	7.4	-361.749343	12.4	-361.756943	18.0	-361.756570
2.4	-361.632797	7.6	-361.750241	12.6	-361.757109	19.0	-361.756065
2.6	-361.654730	7.8	-361.750973	12.8	-361.757263	20.0	-361.755677
2.8	-361.670624	8.0	-361.751567	13.0	-361.757404	21.0	-361.755400
3.0	-361.682173	8.2	-361.752051	13.2	-361.757531	22.0	-361.755204
3.2	-361.690721	8.4	-361.752453	13.4	-361.757642	23.0	-361.755063
3.4	-361.697251	8.6	-361.752793	13.6	-361.757738	24.0	-361.754958
3.6	-361.702464	8.8	-361.753091	13.8	-361.757817	25.0	-361.754879
3.8	-361.706846	9.0	-361.753354	14.0	-361.757880	26.0	-361.754816
4.0	-361.710713	9.2	-361.753594	14.2	-361.757926	27.0	-361.754767
4.2	-361.714238	9.4	-361.753821	14.4	-361.757956	28.0	-361.754727
4.4	-361.717498	9.6	-361.754038	14.6	-361.757970	29.0	-361.754694
4.6	-361.720521	9.8	-361.754251	14.8	-361.757967	30.0	-361.754667
4.8	-361.723332	10.0	-361.754464	15.0	-361.757949	50.0	-361.754517
5.2	-361.728516	10.2	-361.754677	15.2	-361.757917	100.0	-361.754498
5.4	-361.730960	10.4	-361.754891	15.4	-361.757870	200.0	-361.754497
5.6	-361.733342	10.6	-361.755107	15.6	-361.757811	500.0	-361.754497
5.8	-361.735655	10.8	-361.755324	15.8	-361.757740		
6.0	-361.737882	11.0	-361.755540	16.0	-361.757658		
6.2	-361.740000	11.2	-361.755756	16.6	-361.757364		
6.4	-361.741987	11.4	-361.755969	16.8	-361.757255		

Table S7: Total IH-FS-CCSD (2,0)/unANO-RCC+ energies for the $7^1\Sigma^+$ state of $NaMg^+$.

R (Å)	E (a.u.)	R (Å)	E (a.u.)	R (Å)	E (a.u.)	R (Å)	E (a.u.)
1.4	-361.292715	6.6	-361.733741	11.6	-361.749950	17.0	-361.752613
1.6	-361.426267	6.8	-361.735214	11.8	-361.750160	17.2	-361.752629
1.8	-361.503964	7.0	-361.736563	12.0	-361.750359	17.4	-361.752641
2.0	-361.555765	7.2	-361.737797	12.2	-361.750545	17.6	-361.752648
2.2	-361.590481	7.4	-361.738929	12.4	-361.750720	18.0	-361.752653
2.4	-361.615141	7.6	-361.739968	12.6	-361.750886	19.0	-361.752614
2.6	-361.634539	7.8	-361.740924	12.8	-361.751041	20.0	-361.752529
2.8	-361.651688	8.0	-361.741805	13.0	-361.751187	21.0	-361.752430
3.0	-361.664659	8.2	-361.742618	13.2	-361.751323	22.0	-361.752336
3.2	-361.674625	8.4	-361.743370	13.4	-361.751451	23.0	-361.752254
3.4	-361.682504	8.6	-361.744064	13.6	-361.751570	24.0	-361.752185
3.6	-361.688937	8.8	-361.744706	13.8	-361.751682	25.0	-361.752129
3.8	-361.694363	9.0	-361.745299	14.0	-361.751785	26.0	-361.752083
4.0	-361.699068	9.2	-361.745846	14.2	-361.751882	27.0	-361.752044
4.2	-361.703231	9.4	-361.746352	14.4	-361.751971	28.0	-361.752012
4.4	-361.706975	9.6	-361.746818	14.6	-361.752053	29.0	-361.751985
4.6	-361.710388	9.8	-361.747248	14.8	-361.752129	30.0	-361.751962
4.8	-361.713536	10.0	-361.747645	15.0	-361.752198	50.0	-361.751812
5.2	-361.719215	10.2	-361.748012	15.2	-361.752264	100.0	-361.751786
5.4	-361.721785	10.4	-361.748352	15.4	-361.752324	200.0	-361.751788
5.6	-361.724184	10.6	-361.748667	15.6	-361.752378	500.0	-361.751789
5.8	-361.726413	10.8	-361.748960	15.8	-361.752426		
6.0	-361.728477	11.0	-361.749232	16.0	-361.752470		
6.2	-361.730381	11.2	-361.749487	16.6	-361.752568		
6.4	-361.732133	11.4	-361.749726	16.8	-361.752593		

Table S8: Total IH-FS-CCSD (2,0)/unANO-RCC+ energies for the $1^3\Sigma^+$ state of $NaMg^+$.

R (Å)	E (a.u.)	R (Å)	E (a.u.)	R (Å)	E (a.u.)	R (Å)	E (a.u.)
1.4	-361.516593	6.6	-361.881894	11.6	-361.871503	17.0	-361.871189
1.6	-361.647417	6.8	-361.880457	11.8	-361.871475	17.2	-361.871186
1.8	-361.724109	7.0	-361.879155	12.0	-361.871449	17.4	-361.871182
2.0	-361.778251	7.2	-361.877987	12.2	-361.871426	17.6	-361.871179
2.2	-361.818797	7.4	-361.876952	12.4	-361.871405	18.0	-361.871173
2.4	-361.848666	7.6	-361.876045	12.6	-361.871385	19.0	-361.871160
2.6	-361.869924	7.8	-361.875261	12.8	-361.871368	20.0	-361.871150
2.8	-361.884551	8.0	-361.874592	13.0	-361.871351	21.0	-361.871143
3.0	-361.894272	8.2	-361.874029	13.2	-361.871336	22.0	-361.871137
3.2	-361.900438	8.4	-361.873562	13.4	-361.871323	23.0	-361.871132
3.4	-361.904046	8.6	-361.873179	13.6	-361.871310	24.0	-361.871129
3.6	-361.905823	8.8	-361.872867	13.8	-361.871298	25.0	-361.871126
3.8	-361.906297	9.0	-361.872615	14.0	-361.871287	26.0	-361.871123
4.0	-361.905850	9.2	-361.872411	14.2	-361.871277	27.0	-361.871121
4.2	-361.904761	9.4	-361.872245	14.4	-361.871268	28.0	-361.871119
4.4	-361.903231	9.6	-361.872110	14.6	-361.871259	29.0	-361.871118
4.6	-361.901407	9.8	-361.871999	14.8	-361.871251	30.0	-361.871117
4.8	-361.899401	10.0	-361.871906	15.0	-361.871243	50.0	-361.871110
5.2	-361.895146	10.2	-361.871828	15.2	-361.871236	100.0	-361.871109
5.4	-361.893011	10.4	-361.871761	15.4	-361.871229	200.0	-361.871109
5.6	-361.890920	10.6	-361.871703	15.6	-361.871223	500.0	-361.871109
5.8	-361.888902	10.8	-361.871653	15.8	-361.871217		
6.0	-361.886977	11.0	-361.871608	16.0	-361.871212		
6.2	-361.885160	11.2	-361.871569	16.6	-361.871198		
6.4	-361.883463	11.4	-361.871534	16.8	-361.871193		

Table S9: Total IH-FS-CCSD (2,0)/unANO-RCC+ energies for the $2^3\Sigma^+$ state of $NaMg^+$.

R (Å)	E (a.u.)	R (Å)	E (a.u.)	R (Å)	E (a.u.)	R (Å)	E (a.u.)
1.4	-361.397290	6.6	-361.863260	11.6	-361.865602	17.0	-361.864818
1.6	-361.533160	6.8	-361.864131	11.8	-361.865540	17.2	-361.864807
1.8	-361.615417	7.0	-361.864853	12.0	-361.865483	17.4	-361.864796
2.0	-361.673113	7.2	-361.865442	12.2	-361.865431	17.6	-361.864785
2.2	-361.714581	7.4	-361.865911	12.4	-361.865382	18.0	-361.864765
2.4	-361.744128	7.6	-361.866275	12.6	-361.865337	19.0	-361.864724
2.6	-361.765241	7.8	-361.866545	12.8	-361.865295	20.0	-361.864690
2.8	-361.780668	8.0	-361.866731	13.0	-361.865256	21.0	-361.864664
3.0	-361.792390	8.2	-361.866845	13.2	-361.865220	22.0	-361.864642
3.2	-361.801732	8.4	-361.866898	13.4	-361.865186	23.0	-361.864623
3.4	-361.809530	8.6	-361.866899	13.6	-361.865154	24.0	-361.864608
3.6	-361.816286	8.8	-361.866858	13.8	-361.865124	25.0	-361.864596
3.8	-361.822298	9.0	-361.866789	14.0	-361.865095	26.0	-361.864585
4.0	-361.827732	9.2	-361.866700	14.2	-361.865067	27.0	-361.864576
4.2	-361.832675	9.4	-361.866600	14.4	-361.865041	28.0	-361.864568
4.4	-361.837171	9.6	-361.866494	14.6	-361.865017	29.0	-361.864561
4.6	-361.841245	9.8	-361.866391	14.8	-361.864994	30.0	-361.864556
4.8	-361.844913	10.0	-361.866284	15.0	-361.864972	50.0	-361.864514
5.2	-361.851102	10.2	-361.866180	15.2	-361.864957	100.0	-361.864505
5.4	-361.853664	10.4	-361.866082	15.4	-361.864938	200.0	-361.864504
5.6	-361.855907	10.6	-361.865988	15.6	-361.864920	500.0	-361.864504
5.8	-361.857857	10.8	-361.865901	15.8	-361.864903		
6.0	-361.859542	11.0	-361.865819	16.0	-361.864887		
6.2	-361.860988	11.2	-361.865743	16.6	-361.864843		
6.4	-361.862219	11.4	-361.865672	16.8	-361.864830		

Table S10: Total IH-FS-CCSD (2,0)/unANO-RCC+ energies for the $3^3\Sigma^+$ state of $NaMg^+$.

R (Å)	E (a.u.)	R (Å)	E (a.u.)	R (Å)	E (a.u.)	R (Å)	E (a.u.)
1.4	-361.346635	6.6	-361.797550	11.6	-361.798036	17.0	-361.795171
1.6	-361.481655	6.8	-361.798835	11.8	-361.797778	17.2	-361.795139
1.8	-361.561064	7.0	-361.799894	12.0	-361.797540	17.4	-361.795109
2.0	-361.616382	7.2	-361.800748	12.2	-361.797321	17.6	-361.795080
2.2	-361.654690	7.4	-361.801417	12.4	-361.797120	18.0	-361.795027
2.4	-361.682730	7.6	-361.801920	12.6	-361.796936	19.0	-361.794917
2.6	-361.702503	7.8	-361.802276	12.8	-361.796768	20.0	-361.794831
2.8	-361.716314	8.0	-361.802503	13.0	-361.796615	21.0	-361.794762
3.0	-361.726015	8.2	-361.802616	13.2	-361.796474	22.0	-361.794707
3.2	-361.733017	8.4	-361.802630	13.4	-361.796346	23.0	-361.794663
3.4	-361.738362	8.6	-361.802560	13.6	-361.796229	24.0	-361.794626
3.6	-361.742830	8.8	-361.802421	13.8	-361.796122	25.0	-361.794595
3.8	-361.747075	9.0	-361.802219	14.0	-361.796023	26.0	-361.794569
4.0	-361.751641	9.2	-361.801968	14.2	-361.795932	27.0	-361.794548
4.2	-361.756707	9.4	-361.801678	14.4	-361.795849	28.0	-361.794529
4.4	-361.761984	9.6	-361.801358	14.6	-361.795772	29.0	-361.794514
4.6	-361.767125	9.8	-361.801018	14.8	-361.795700	30.0	-361.794500
4.8	-361.771931	10.0	-361.800666	15.0	-361.795634	50.0	-361.794407
5.2	-361.780290	10.2	-361.800308	15.2	-361.795572	100.0	-361.794387
5.4	-361.783824	10.4	-361.799950	15.4	-361.795515	200.0	-361.794384
5.6	-361.786955	10.6	-361.799597	15.6	-361.795462	500.0	-361.794384
5.8	-361.789712	10.8	-361.799255	15.8	-361.795412		
6.0	-361.792124	11.0	-361.798925	16.0	-361.795365		
6.2	-361.794217	11.2	-361.798612	16.6	-361.795242		
6.4	-361.796018	11.4	-361.798316	16.8	-361.795206		

Table S11: Total IH-FS-CCSD (2,0)/unANO-RCC+ energies for the $4^3\Sigma^+$ state of $NaMg^+$.

R (Å)	E (a.u.)	R (Å)	E (a.u.)	R (Å)	E (a.u.)	R (Å)	E (a.u.)
1.4	-361.300179	6.6	-361.763521	11.6	-361.779462	17.0	-361.777113
1.6	-361.435350	6.8	-361.764623	11.8	-361.779449	17.2	-361.777066
1.8	-361.515953	7.0	-361.765727	12.0	-361.779409	17.4	-361.777023
2.0	-361.573051	7.2	-361.766829	12.2	-361.779346	17.6	-361.776983
2.2	-361.615659	7.4	-361.767922	12.4	-361.779265	18.0	-361.776910
2.4	-361.647222	7.6	-361.768996	12.6	-361.779170	19.0	-361.776764
2.6	-361.670413	7.8	-361.770043	12.8	-361.779063	20.0	-361.776659
2.8	-361.687650	8.0	-361.771054	13.0	-361.778949	21.0	-361.776579
3.0	-361.700907	8.2	-361.772021	13.2	-361.778829	22.0	-361.776519
3.2	-361.711611	8.4	-361.772937	13.4	-361.778707	23.0	-361.776472
3.4	-361.720663	8.6	-361.773798	13.6	-361.778584	24.0	-361.776435
3.6	-361.728507	8.8	-361.774596	13.8	-361.778462	25.0	-361.776406
3.8	-361.735203	9.0	-361.775334	14.0	-361.778342	26.0	-361.776382
4.0	-361.740568	9.2	-361.776008	14.2	-361.778226	27.0	-361.776363
4.2	-361.744584	9.4	-361.776618	14.4	-361.778114	28.0	-361.776347
4.4	-361.747596	9.6	-361.777163	14.6	-361.778006	29.0	-361.776334
4.6	-361.749983	9.8	-361.777644	14.8	-361.777904	30.0	-361.776323
4.8	-361.751973	10.0	-361.778062	15.0	-361.777807	50.0	-361.776259
5.2	-361.755219	10.2	-361.778418	15.2	-361.777716	100.0	-361.776250
5.4	-361.756597	10.4	-361.778716	15.4	-361.777629	200.0	-361.776250
5.6	-361.757868	10.6	-361.778958	15.6	-361.777548	500.0	-361.776250
5.8	-361.759062	10.8	-361.779148	15.8	-361.777473		
6.0	-361.760205	11.0	-361.779289	16.0	-361.777402		
6.2	-361.761319	11.2	-361.779385	16.6	-361.777216		
6.4	-361.762421	11.4	-361.779440	16.8	-361.777163		

Table S12: Total IH-FS-CCSD (2,0)/unANO-RCC+ energies for the $5^3\Sigma^+$ state of $NaMg^+$.

R (Å)	E (a.u.)	R (Å)	E (a.u.)	R (Å)	E (a.u.)	R (Å)	E (a.u.)
1.4	-361.271184	6.6	-361.738305	11.6	-361.759376	17.0	-361.757787
1.6	-361.408540	6.8	-361.739428	11.8	-361.759631	17.2	-361.757596
1.8	-361.490391	7.0	-361.740434	12.0	-361.759849	17.4	-361.757410
2.0	-361.547722	7.2	-361.741372	12.2	-361.760032	17.6	-361.757231
2.2	-361.589738	7.4	-361.742322	12.4	-361.760181	18.0	-361.756895
2.4	-361.620610	7.6	-361.743384	12.6	-361.760297	19.0	-361.756208
2.6	-361.643335	7.8	-361.744602	12.8	-361.760383	20.0	-361.755733
2.8	-361.660177	8.0	-361.745905	13.0	-361.760439	21.0	-361.755420
3.0	-361.672816	8.2	-361.747203	13.2	-361.760467	22.0	-361.755211
3.2	-361.682474	8.4	-361.748450	13.4	-361.760468	23.0	-361.755065
3.4	-361.690029	8.6	-361.749624	13.6	-361.760444	24.0	-361.754959
3.6	-361.696127	8.8	-361.750720	13.8	-361.760396	25.0	-361.754879
3.8	-361.701236	9.0	-361.751737	14.0	-361.760326	26.0	-361.754816
4.0	-361.705681	9.2	-361.752679	14.2	-361.760235	27.0	-361.754767
4.2	-361.709667	9.4	-361.753549	14.4	-361.760125	28.0	-361.754727
4.4	-361.713314	9.6	-361.754352	14.6	-361.759998	29.0	-361.754694
4.6	-361.716686	9.8	-361.755091	14.8	-361.759856	30.0	-361.754667
4.8	-361.719815	10.0	-361.755770	15.0	-361.759700	50.0	-361.754517
5.2	-361.725392	10.2	-361.756392	15.2	-361.759534	100.0	-361.754498
5.4	-361.727853	10.4	-361.756960	15.4	-361.759356	200.0	-361.754497
5.6	-361.730099	10.6	-361.757477	15.6	-361.759171	500.0	-361.754497
5.8	-361.732134	10.8	-361.757945	15.8	-361.758979		
6.0	-361.733962	11.0	-361.758367	16.0	-361.758782		
6.2	-361.735591	11.2	-361.758744	16.6	-361.758181		
6.4	-361.737034	11.4	-361.759078	16.8	-361.757983		

Table S13: Total IH-FS-CCSD (2,0)/unANO-RCC+ energies for the $6^3\Sigma^+$ state of $NaMg^+$.

R (Å)	E (a.u.)	R (Å)	E (a.u.)	R (Å)	E (a.u.)	R (Å)	E (a.u.)
1.4	-361.266151	6.6	-361.731337	11.6	-361.746511	17.0	-361.750846
1.6	-361.402172	6.8	-361.733597	11.8	-361.746717	17.2	-361.750861
1.8	-361.483608	7.0	-361.735707	12.0	-361.746932	17.4	-361.750863
2.0	-361.540152	7.2	-361.737628	12.2	-361.747156	17.6	-361.750854
2.2	-361.581245	7.4	-361.739298	12.4	-361.747385	18.0	-361.750802
2.4	-361.611097	7.6	-361.740634	12.6	-361.747617	19.0	-361.750510
2.6	-361.632676	7.8	-361.741611	12.8	-361.747851	20.0	-361.750067
2.8	-361.648456	8.0	-361.742318	13.0	-361.748084	21.0	-361.749581
3.0	-361.660412	8.2	-361.742860	13.2	-361.748313	22.0	-361.749126
3.2	-361.669998	8.4	-361.743298	13.4	-361.748538	23.0	-361.748730
3.4	-361.678189	8.6	-361.743665	13.6	-361.748757	24.0	-361.748394
3.6	-361.685460	8.8	-361.743977	13.8	-361.748969	25.0	-361.748111
3.8	-361.691861	9.0	-361.744247	14.0	-361.749171	26.0	-361.747872
4.0	-361.697295	9.2	-361.744481	14.2	-361.749364	27.0	-361.747669
4.2	-361.701738	9.4	-361.744688	14.4	-361.749547	28.0	-361.747494
4.4	-361.705268	9.6	-361.744872	14.6	-361.749719	29.0	-361.747344
4.6	-361.708024	9.8	-361.745040	14.8	-361.749880	30.0	-361.747213
4.8	-361.710183	10.0	-361.745196	15.0	-361.750029	50.0	-361.746279
5.2	-361.713833	10.2	-361.745346	15.2	-361.750165	100.0	-361.746097
5.4	-361.716089	10.4	-361.745493	15.4	-361.750291	200.0	-361.746082
5.6	-361.718654	10.6	-361.745642	15.6	-361.750403	500.0	-361.746080
5.8	-361.721294	10.8	-361.745797	15.8	-361.750504		
6.0	-361.723912	11.0	-361.745959	16.0	-361.750592		
6.2	-361.726473	11.2	-361.746132	16.6	-361.750782		
6.4	-361.728954	11.4	-361.746315	16.8	-361.750820		

Table S14: Total IH-FS-CCSD (2,0)/unANO-RCC+ energies for the $1^1\Pi$ state of $NaMg^+$.

R (Å)	E (a.u.)	R (Å)	E (a.u.)	R (Å)	E (a.u.)	R (Å)	E (a.u.)
1.4	-361.468955	6.6	-361.806211	11.6	-361.803871	17.0	-361.803983
1.6	-361.598404	6.8	-361.805851	11.8	-361.803876	17.2	-361.803986
1.8	-361.670322	7.0	-361.805526	12.0	-361.803880	17.4	-361.803988
2.0	-361.717312	7.2	-361.805238	12.2	-361.803885	17.6	-361.803991
2.2	-361.750128	7.4	-361.804986	12.4	-361.803890	18.0	-361.803995
2.4	-361.772958	7.6	-361.804769	12.6	-361.803895	19.0	-361.804006
2.6	-361.788311	7.8	-361.804584	12.8	-361.803900	20.0	-361.804015
2.8	-361.798211	8.0	-361.804429	13.0	-361.803905	21.0	-361.804022
3.0	-361.804306	8.2	-361.804300	13.2	-361.803910	22.0	-361.804029
3.2	-361.807857	8.4	-361.804195	13.4	-361.803915	23.0	-361.804035
3.4	-361.809776	8.6	-361.804110	13.6	-361.803920	24.0	-361.804040
3.6	-361.810701	8.8	-361.804043	13.8	-361.803925	25.0	-361.804044
3.8	-361.811055	9.0	-361.803990	14.0	-361.803929	26.0	-361.804048
4.0	-361.811101	9.2	-361.803949	14.2	-361.803933	27.0	-361.804051
4.2	-361.810980	9.4	-361.803919	14.4	-361.803938	28.0	-361.804054
4.4	-361.810762	9.6	-361.803896	14.6	-361.803942	29.0	-361.804057
4.6	-361.810476	9.8	-361.803880	14.8	-361.803946	30.0	-361.804059
4.8	-361.810136	10.0	-361.803870	15.0	-361.803950	50.0	-361.804077
5.2	-361.809332	10.2	-361.803863	15.2	-361.803955	100.0	-361.804082
5.4	-361.808879	10.4	-361.803859	15.4	-361.803958	200.0	-361.804083
5.6	-361.808413	10.6	-361.803858	15.6	-361.803962	500.0	-361.804083
5.8	-361.807942	10.8	-361.803858	15.8	-361.803965		
6.0	-361.807478	11.0	-361.803860	16.0	-361.803968		
6.2	-361.807029	11.2	-361.803863	16.6	-361.803977		
6.4	-361.806605	11.4	-361.803867	16.8	-361.803980		

Table S15: Total IH-FS-CCSD (2,0)/unANO-RCC+ energies for the $2^1\Pi$ state of $NaMg^+$.

R (Å)	E (a.u.)	R (Å)	E (a.u.)	R (Å)	E (a.u.)	R (Å)	E (a.u.)
1.4	-361.392545	6.6	-361.790864	11.6	-361.794166	17.0	-361.794265
1.6	-361.524413	6.8	-361.791420	11.8	-361.794170	17.2	-361.794268
1.8	-361.600100	7.0	-361.791911	12.0	-361.794174	17.4	-361.794270
2.0	-361.651821	7.2	-361.792330	12.2	-361.794178	17.6	-361.794273
2.2	-361.689506	7.4	-361.792688	12.4	-361.794182	18.0	-361.794278
2.4	-361.717082	7.6	-361.792992	12.6	-361.794186	19.0	-361.794290
2.6	-361.737098	7.8	-361.793240	12.8	-361.794190	20.0	-361.794300
2.8	-361.751535	8.0	-361.793444	13.0	-361.794194	21.0	-361.794309
3.0	-361.761904	8.2	-361.793606	13.2	-361.794198	22.0	-361.794317
3.2	-361.769296	8.4	-361.793737	13.4	-361.794202	23.0	-361.794323
3.4	-361.774478	8.6	-361.793838	13.6	-361.794206	24.0	-361.794329
3.6	-361.778007	8.8	-361.793917	13.8	-361.794210	25.0	-361.794334
3.8	-361.780318	9.0	-361.793977	14.0	-361.794214	26.0	-361.794339
4.0	-361.781774	9.2	-361.794023	14.2	-361.794217	27.0	-361.794343
4.2	-361.782684	9.4	-361.794057	14.4	-361.794221	28.0	-361.794347
4.4	-361.783300	9.6	-361.794083	14.6	-361.794225	29.0	-361.794350
4.6	-361.783812	9.8	-361.794102	14.8	-361.794229	30.0	-361.794353
4.8	-361.784342	10.0	-361.794115	15.0	-361.794232	50.0	-361.794376
5.2	-361.785646	10.2	-361.794127	15.2	-361.794236	100.0	-361.794383
5.4	-361.786411	10.4	-361.794136	15.4	-361.794239	200.0	-361.794384
5.6	-361.787214	10.6	-361.794142	15.6	-361.794243	500.0	-361.794384
5.8	-361.788021	10.8	-361.794148	15.8	-361.794246		
6.0	-361.788808	11.0	-361.794153	16.0	-361.794249		
6.2	-361.789553	11.2	-361.794158	16.6	-361.794259		
6.4	-361.790242	11.4	-361.794162	16.8	-361.794262		

Table S16: Total IH-FS-CCSD (2,0)/unANO-RCC+ energies for the $3^1\Pi$ state of $NaMg^+$.

R (Å)	E (a.u.)	R (Å)	E (a.u.)	R (Å)	E (a.u.)	R (Å)	E (a.u.)
1.4	-361.321829	6.6	-361.752467	11.6	-361.754741	17.0	-361.752497
1.6	-361.457358	6.8	-361.753038	11.8	-361.754605	17.2	-361.752466
1.8	-361.535082	7.0	-361.753565	12.0	-361.754471	17.4	-361.752438
2.0	-361.587193	7.2	-361.754038	12.2	-361.754339	17.6	-361.752411
2.2	-361.624376	7.4	-361.754461	12.4	-361.754210	18.0	-361.752362
2.4	-361.651256	7.6	-361.754829	12.6	-361.754084	19.0	-361.752262
2.6	-361.670793	7.8	-361.755142	12.8	-361.753962	20.0	-361.752185
2.8	-361.685247	8.0	-361.755400	13.0	-361.753844	21.0	-361.752123
3.0	-361.696337	8.2	-361.755606	13.2	-361.753732	22.0	-361.752074
3.2	-361.705277	8.4	-361.755763	13.4	-361.753624	23.0	-361.752034
3.4	-361.712827	8.6	-361.755875	13.6	-361.753522	24.0	-361.752000
3.6	-361.719400	8.8	-361.755943	13.8	-361.753425	25.0	-361.751972
3.8	-361.725178	9.0	-361.755976	14.0	-361.753333	26.0	-361.751949
4.0	-361.730223	9.2	-361.755976	14.2	-361.753247	27.0	-361.751929
4.2	-361.734548	9.4	-361.755948	14.4	-361.753167	28.0	-361.751913
4.4	-361.738168	9.6	-361.755897	14.6	-361.753091	29.0	-361.751898
4.6	-361.741119	9.8	-361.755827	14.8	-361.753021	30.0	-361.751886
4.8	-361.743471	10.0	-361.755739	15.0	-361.752955	50.0	-361.751804
5.2	-361.746776	10.2	-361.755637	15.2	-361.752889	100.0	-361.751788
5.4	-361.747933	10.4	-361.755525	15.4	-361.752832	200.0	-361.751786
5.6	-361.748901	10.6	-361.755405	15.6	-361.752778	500.0	-361.751786
5.8	-361.749747	10.8	-361.755278	15.8	-361.752729		
6.0	-361.750503	11.0	-361.755148	16.0	-361.752683		
6.2	-361.751202	11.2	-361.755014	16.6	-361.752565		
6.4	-361.751856	11.4	-361.754878	16.8	-361.752529		

Table S17: Total IH-FS-CCSD (2,0)/unANO-RCC+ energies for the $1^3\Pi$ state of $NaMg^+$.

R (Å)	E (a.u.)	R (Å)	E (a.u.)	R (Å)	E (a.u.)	R (Å)	E (a.u.)
1.4	-361.551282	6.6	-361.864105	11.6	-361.864280	17.0	-361.864414
1.6	-361.679250	6.8	-361.864079	11.8	-361.864288	17.2	-361.864417
1.8	-361.749426	7.0	-361.864065	12.0	-361.864296	17.4	-361.864419
2.0	-361.793972	7.2	-361.864059	12.2	-361.864304	17.6	-361.864422
2.2	-361.823685	7.4	-361.864058	12.4	-361.864311	18.0	-361.864426
2.4	-361.843282	7.6	-361.864062	12.6	-361.864318	19.0	-361.864436
2.6	-361.855678	7.8	-361.864069	12.8	-361.864324	20.0	-361.864445
2.8	-361.863067	8.0	-361.864078	13.0	-361.864331	21.0	-361.864452
3.0	-361.867114	8.2	-361.864089	13.2	-361.864337	22.0	-361.864458
3.2	-361.869025	8.4	-361.864100	13.4	-361.864343	23.0	-361.864463
3.4	-361.869630	8.6	-361.864112	13.6	-361.864348	24.0	-361.864467
3.6	-361.869482	8.8	-361.864124	13.8	-361.864353	25.0	-361.864471
3.8	-361.868947	9.0	-361.864137	14.0	-361.864359	26.0	-361.864474
4.0	-361.868252	9.2	-361.864150	14.2	-361.864363	27.0	-361.864477
4.2	-361.867532	9.4	-361.864162	14.4	-361.864368	28.0	-361.864480
4.4	-361.866859	9.6	-361.864175	14.6	-361.864373	29.0	-361.864482
4.6	-361.866268	9.8	-361.864187	14.8	-361.864377	30.0	-361.864484
4.8	-361.865768	10.0	-361.864199	15.0	-361.864381	50.0	-361.864499
5.2	-361.865025	10.2	-361.864211	15.2	-361.864385	100.0	-361.864502
5.4	-361.864764	10.4	-361.864222	15.4	-361.864389	200.0	-361.864503
5.6	-361.864561	10.6	-361.864233	15.6	-361.864393	500.0	-361.864503
5.8	-361.864406	10.8	-361.864243	15.8	-361.864396		
6.0	-361.864289	11.0	-361.864253	16.0	-361.864399		
6.2	-361.864204	11.2	-361.864262	16.6	-361.864409		
6.4	-361.864145	11.4	-361.864271	16.8	-361.864412		

Table S18: Total IH-FS-CCSD (2,0)/unANO-RCC+ energies for the $2^3\Pi$ state of $NaMg^+$.

R (Å)	E (a.u.)	R (Å)	E (a.u.)	R (Å)	E (a.u.)	R (Å)	E (a.u.)
1.4	-361.371783	6.6	-361.794124	11.6	-361.794177	17.0	-361.794265
1.6	-361.504839	6.8	-361.794256	11.8	-361.794178	17.2	-361.794268
1.8	-361.581943	7.0	-361.794346	12.0	-361.794180	17.4	-361.794270
2.0	-361.634849	7.2	-361.794396	12.2	-361.794183	17.6	-361.794273
2.2	-361.673445	7.4	-361.794422	12.4	-361.794185	18.0	-361.794278
2.4	-361.701702	7.6	-361.794432	12.6	-361.794189	19.0	-361.794290
2.6	-361.722226	7.8	-361.794426	12.8	-361.794192	20.0	-361.794300
2.8	-361.737157	8.0	-361.794410	13.0	-361.794195	21.0	-361.794309
3.0	-361.748203	8.2	-361.794387	13.2	-361.794199	22.0	-361.794317
3.2	-361.756619	8.4	-361.794365	13.4	-361.794203	23.0	-361.794323
3.4	-361.763257	8.6	-361.794339	13.6	-361.794206	24.0	-361.794329
3.6	-361.768667	8.8	-361.794314	13.8	-361.794210	25.0	-361.794334
3.8	-361.773190	9.0	-361.794290	14.0	-361.794214	26.0	-361.794339
4.0	-361.777031	9.2	-361.794268	14.2	-361.794218	27.0	-361.794343
4.2	-361.780307	9.4	-361.794248	14.4	-361.794221	28.0	-361.794347
4.4	-361.783093	9.6	-361.794231	14.6	-361.794225	29.0	-361.794350
4.6	-361.785442	9.8	-361.794217	14.8	-361.794229	30.0	-361.794353
4.8	-361.787400	10.0	-361.794204	15.0	-361.794232	50.0	-361.794376
5.2	-361.790317	10.2	-361.794196	15.2	-361.794236	100.0	-361.794383
5.4	-361.791358	10.4	-361.794189	15.4	-361.794239	200.0	-361.794384
5.6	-361.792179	10.6	-361.794182	15.6	-361.794243	500.0	-361.794384
5.8	-361.792815	10.8	-361.794179	15.8	-361.794246		
6.0	-361.793301	11.0	-361.794176	16.0	-361.794249		
6.2	-361.793666	11.2	-361.794176	16.6	-361.794259		
6.4	-361.793934	11.4	-361.794176	16.8	-361.794262		

Table S19: Total IH-FS-CCSD (2,0)/unANO-RCC+ energies for the $3^3\Pi$ state of $NaMg^+$.

R (Å)	E (a.u.)	R (Å)	E (a.u.)	R (Å)	E (a.u.)	R (Å)	E (a.u.)
1.4	-361.357355	6.6	-361.746008	11.6	-361.753107	17.0	-361.748649
1.6	-361.487705	6.8	-361.747456	11.8	-361.752910	17.2	-361.748560
1.8	-361.559057	7.0	-361.748732	12.0	-361.752710	17.4	-361.748475
2.0	-361.605821	7.2	-361.749843	12.2	-361.752508	17.6	-361.748394
2.2	-361.639901	7.4	-361.750806	12.4	-361.752303	18.0	-361.748245
2.4	-361.665586	7.6	-361.751629	12.6	-361.752098	19.0	-361.747930
2.6	-361.684977	7.8	-361.752322	12.8	-361.751893	20.0	-361.747677
2.8	-361.699527	8.0	-361.752897	13.0	-361.751690	21.0	-361.747468
3.0	-361.710378	8.2	-361.753365	13.2	-361.751488	22.0	-361.747292
3.2	-361.718412	8.4	-361.753736	13.4	-361.751288	23.0	-361.747142
3.4	-361.724276	8.6	-361.754021	13.6	-361.751093	24.0	-361.747013
3.6	-361.728437	8.8	-361.754229	13.8	-361.750901	25.0	-361.746901
3.8	-361.731235	9.0	-361.754369	14.0	-361.750714	26.0	-361.746804
4.0	-361.732929	9.2	-361.754451	14.2	-361.750532	27.0	-361.746720
4.2	-361.733735	9.4	-361.754484	14.4	-361.750356	28.0	-361.746647
4.4	-361.733834	9.6	-361.754473	14.6	-361.750185	29.0	-361.746583
4.6	-361.733388	9.8	-361.754426	14.8	-361.750021	30.0	-361.746527
4.8	-361.732539	10.0	-361.754350	15.0	-361.749864	50.0	-361.746138
5.2	-361.730835	10.2	-361.754247	15.2	-361.749713	100.0	-361.746082
5.4	-361.733180	10.4	-361.754123	15.4	-361.749569	200.0	-361.746080
5.6	-361.735817	10.6	-361.753983	15.6	-361.749432	500.0	-361.746080
5.8	-361.738282	10.8	-361.753827	15.8	-361.749302		
6.0	-361.740525	11.0	-361.753660	16.0	-361.749178		
6.2	-361.742555	11.2	-361.753483	16.6	-361.748844		
6.4	-361.744381	11.4	-361.753298	16.8	-361.748744		

Table S20: Total IH-FS-CCSD (2,0)/unANO-RCC+ energies for the $1^1\Delta$ state of $NaMg^+$.

R (Å)	E (a.u.)	R (Å)	E (a.u.)	R (Å)	E (a.u.)	R (Å)	E (a.u.)
1.4	-361.414044	6.6	-361.746748	11.6	-361.750352	17.0	-361.751289
1.6	-361.540576	6.8	-361.747022	11.8	-361.750415	17.2	-361.751305
1.8	-361.607774	7.0	-361.747278	12.0	-361.750475	17.4	-361.751321
2.0	-361.649739	7.2	-361.747517	12.2	-361.750531	17.6	-361.751336
2.2	-361.678453	7.4	-361.747741	12.4	-361.750585	18.0	-361.751365
2.4	-361.698600	7.6	-361.747951	12.6	-361.750635	19.0	-361.751426
2.6	-361.712584	7.8	-361.748148	12.8	-361.750683	20.0	-361.751476
2.8	-361.722106	8.0	-361.748334	13.0	-361.750729	21.0	-361.751517
3.0	-361.728502	8.2	-361.748508	13.2	-361.750772	22.0	-361.751552
3.2	-361.732786	8.4	-361.748673	13.4	-361.750813	23.0	-361.751581
3.4	-361.735681	8.6	-361.748829	13.6	-361.750852	24.0	-361.751605
3.6	-361.737685	8.8	-361.748976	13.8	-361.750889	25.0	-361.751626
3.8	-361.739130	9.0	-361.749114	14.0	-361.750924	26.0	-361.751643
4.0	-361.740230	9.2	-361.749245	14.2	-361.750957	27.0	-361.751659
4.2	-361.741117	9.4	-361.749369	14.4	-361.750989	28.0	-361.751672
4.4	-361.741867	9.6	-361.749486	14.6	-361.751019	29.0	-361.751683
4.6	-361.742525	9.8	-361.749596	14.8	-361.751048	30.0	-361.751693
4.8	-361.743118	10.0	-361.749701	15.0	-361.751076	50.0	-361.751768
5.2	-361.744155	10.2	-361.749799	15.2	-361.751102	100.0	-361.751786
5.4	-361.744614	10.4	-361.749892	15.4	-361.751127	200.0	-361.751788
5.6	-361.745039	10.6	-361.749980	15.6	-361.751150	500.0	-361.751789
5.8	-361.745433	10.8	-361.750063	15.8	-361.751173		
6.0	-361.745798	11.0	-361.750142	16.0	-361.751195		
6.2	-361.746138	11.2	-361.750216	16.6	-361.751254		
6.4	-361.746454	11.4	-361.750286	16.8	-361.751272		
